# Sphingosine kinase 2 inhibition synergises with bortezomib to target myeloma by enhancing endoplasmic reticulum stress

**DOI:** 10.18632/oncotarget.17115

**Published:** 2017-04-14

**Authors:** Craig T. Wallington-Beddoe, Melissa K. Bennett, Kate Vandyke, Lorena Davies, Julia R. Zebol, Paul A.B. Moretti, Melissa R. Pitman, Duncan R. Hewett, Andrew C.W. Zannettino, Stuart M Pitson

**Affiliations:** ^1^ Center for Cancer Biology, University of South Australia, Adelaide, Australia; ^2^ SA Pathology, Adelaide, Australia; ^3^ School of Medicine, University of Adelaide, Australia; ^4^ South Australian Health and Medical Research Institute, Adelaide, Australia

**Keywords:** myeloma, endoplasmic reticulum, proteasome inhibitor, sphingosine kinase

## Abstract

The proteasome inhibitor bortezomib has proven to be invaluable in the treatment of myeloma. By exploiting the inherent high immunoglobulin protein production of malignant plasma cells, bortezomib induces endoplasmic reticulum (ER) stress and the unfolded protein response (UPR), resulting in myeloma cell death. In most cases, however, the disease remains incurable highlighting the need for new therapeutic targets. Sphingosine kinase 2 (SK2) has been proposed as one such therapeutic target for myeloma. Our observations that bortezomib and SK2 inhibitors independently elicited induction of ER stress and the UPR prompted us to examine potential synergy between these agents in myeloma. Targeting SK2 synergistically contributed to ER stress and UPR activation induced by bortezomib, as evidenced by activation of the IRE1 pathway and stress kinases JNK and p38MAPK, thereby resulting in potent synergistic myeloma apoptosis *in vitro*. The combination of bortezomib and SK2 inhibition also exhibited strong *in vivo* synergy and favourable effects on bone disease. Therefore, our studies suggest that perturbations of sphingolipid signalling can synergistically enhance the effects seen with proteasome inhibition, highlighting the potential for the combination of these two modes of increasing ER stress to be formally evaluated in clinical trials for the treatment of myeloma patients.

## INTRODUCTION

Myeloma is a relatively common and incurable malignancy of plasma cells with an age-adjusted incidence of six per 100 000 per year in the USA and Europe [1]. Despite recent advances in therapy and supportive care, the median overall survival is 6 years [1]. In recent years, the introduction of the 26S proteasome inhibitor bortezomib into multi-drug treatment regimes has dramatically improved remission rates, however, disease relapse inevitably occurs [2, 3]. Myeloma cells produce and secrete large quantities of immunoglobulin, which requires folding within the endoplasmic reticulum (ER). The development of ER stress occurs when misfolded proteins accumulate and overwhelm the folding capacity of the ER. This results in the initiation of adaptive homeostatic processes, including expansion of ER protein chaperone and folding capacity, removal of misfolded proteins from the ER for proteasomal degradation, a process termed ER-associated degradation (ERAD), and activation of the so-called unfolded protein response (UPR) [4]. The UPR is initiated when the accumulation of misfolded proteins results in the dissociation of the ER chaperone binding immunoglobulin protein (BiP) from the lumenal domains of the three ER stress transmembrane protein sensors: protein kinase R-like ER kinase (PERK), inositol-requiring kinase 1 (IRE1) and activating transcription factor 6 (ATF6) [4, 5]. From each of these UPR initiating proteins, a cascade of protein interactions ensues, with the net result being an expansion of ER folding capacity, a reduction in protein generation and hence the entry of nascent proteins into the ER, and enhanced clearance of unfolded proteins *via* ERAD. However, if ER stress continues for a prolonged period, the generally pro-survival adaptive measures cease and sustained UPR activation results in expression of the pro-apoptotic transcription factor CHOP (CCAAT/enhancer binding protein (C/EBP) homologous protein) and induction of cell death [6]. By exploiting the sensitivity of myeloma cells to ER stress, proteasome inhibition by bortezomib produces sustained UPR activation that ultimately results in myeloma cell death [7].

Sphingosine kinase 2 (SK2) is one of two SK isoforms that catalyses the phosphorylation of sphingosine to sphingosine 1-phosphate (S1P), a sphingolipid implicated in cancer growth and survival [8]. SK2 is found in discrete subcellular locations including the ER and nucleus, the latter shown to be involved in Myc transcription in acute lymphoblastic leukaemia (ALL) with inhibition of SK2 exhibiting anti-leukaemic efficacy [9, 10]. More recently, inhibition of SK2 has shown some efficacy as a monotherapy in a pre-clinical myeloma study, although the mechanisms for these effects were not well defined [11]. In the current study, we show that SK2 inhibition has anti-myeloma activity by inducing ER stress and activating the UPR. Furthermore, combining SK2 inhibition with low dose bortezomib produced synergistic ER stress and UPR activation that potently induced apoptosis associated with activation of the stress kinases c-Jun N-terminal kinase (JNK) and p38 mitogen-activated protein kinase (p38MAPK) that are known to associate with the IRE1 arm of the UPR [12, 13]. Finally, we found that this dual therapeutic strategy synergistically reduced disease burden in an aggressive immunocompetent murine model of myeloma, raising the potential to progress such a therapeutic strategy towards clinical trials for the treatment of human myeloma.

## RESULTS

### Sphingosine kinase 2 as a target in multiple myeloma

**Figure 1:**
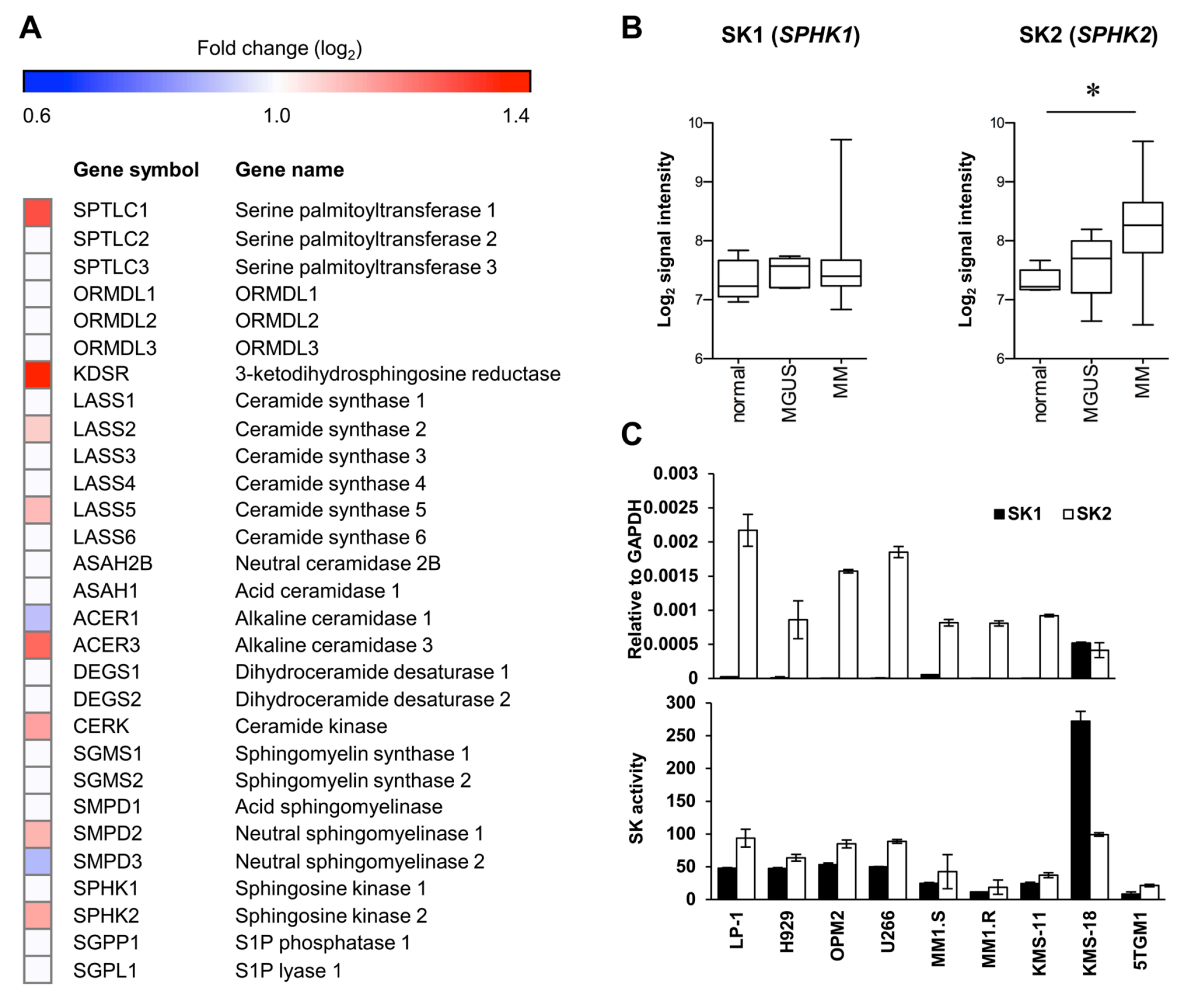
SK2 has higher expression than SK1 in myeloma **A**. Expression (log_2_) of sphingolipid enzymes in the publically available gene expression dataset E-MTAB-363 [14] of purified CD138+ bone marrow plasma cells from normal healthy (*n* = 5), MGUS (*n* = 5) and myeloma (*n* = 155) patients. The heatmap shows log_2_-fold changes where strong evidence existed (*P* < 0.01, Kruskal-Wallis test) for different median gene expression between normal and myeloma patients. All genes analysed are listed, but genes lacking strong evidence for differential expression (*P* > 0.01) are indicated with white boxes. **B**. Analysis of E-MTAB-363 demonstrates no change in SK1 (*SPHK1*) as disease progresses whereas SK2 (*SPHK2*) increases significantly between normal and MM (*P = 0.0004, Kruskal-Wallis test). Signal intensity represents log_2_ gene expression. **C**. SK1 and SK2 gene expression was analysed by RT-qPCR in the indicated human myeloma cell lines (upper) showing greater SK2 expression in all but one cell line examined. Enzyme activity assay for SK1 and SK2 using human myeloma cell lines (lower) reveals SK2 is more active than SK1 in most cell lines. Data are mean±SD of triplicate measurements and are representative of three independent experiments.

Elevated expression of SK2 has been demonstrated previously in newly diagnosed myeloma patient CD138+ cells compared to plasma cells from healthy normal individuals [11]. To assess these findings in more detail, we examined the expression levels of SK2 and other genes involved in sphingolipid biosynthesis, in a different, larger dataset comprised of gene expression data from CD138+ bone marrow plasma cells from newly diagnosed myeloma patients compared to normal healthy controls [14]. Notably, this analysis revealed that numerous genes critical to sphingolipid synthesis and metabolism to S1P were significantly elevated in myeloma, including serine palmitoyl transferase 1, 3-ketodihydrosphingosine reductase, ceramide synthases 2 and 5, sphingomyelinase 2, and alkaline ceramidase 3 (Figure 1A and Supplementary Figure 1), supporting the notion that sphingolipid metabolism is dysregulated in myeloma. Further gene set enrichment analysis failed to find enrichment of gene sets associated with sphingolipid synthesis or metabolism in MGUS or myeloma patients compared to normal healthy controls using this dataset. However, the changes in gene expression of the various enzymes in this pathway indicates a general increase in *de novo* ceramide synthesis, suggesting increased dependency on sphingosine kinases to metabolise the resultant ceramide. While plasma cell expression of SK1 was similar between healthy normal individuals, patients with monoclonal gammopathy of undetermined significance (MGUS) and myeloma, the expression of SK2 was significantly increased (*P* < 0.001, Kruskal-Wallis test) in myeloma patients compared with healthy normal age matched controls (Figure 1B).

Next, we compared the expression levels of SK1 and SK2 in a range of human myeloma cell lines. Consistent with previous studies using other myeloma cell lines [11], both RT-qPCR and isoform-selective enzyme assays revealed much higher SK2 levels compared to SK1 in almost all cell lines examined (Figure 1C). Taken together, these data suggest SK2 is more relevant than SK1 in myeloma and represents a potential therapeutic target.

**Figure 2:**
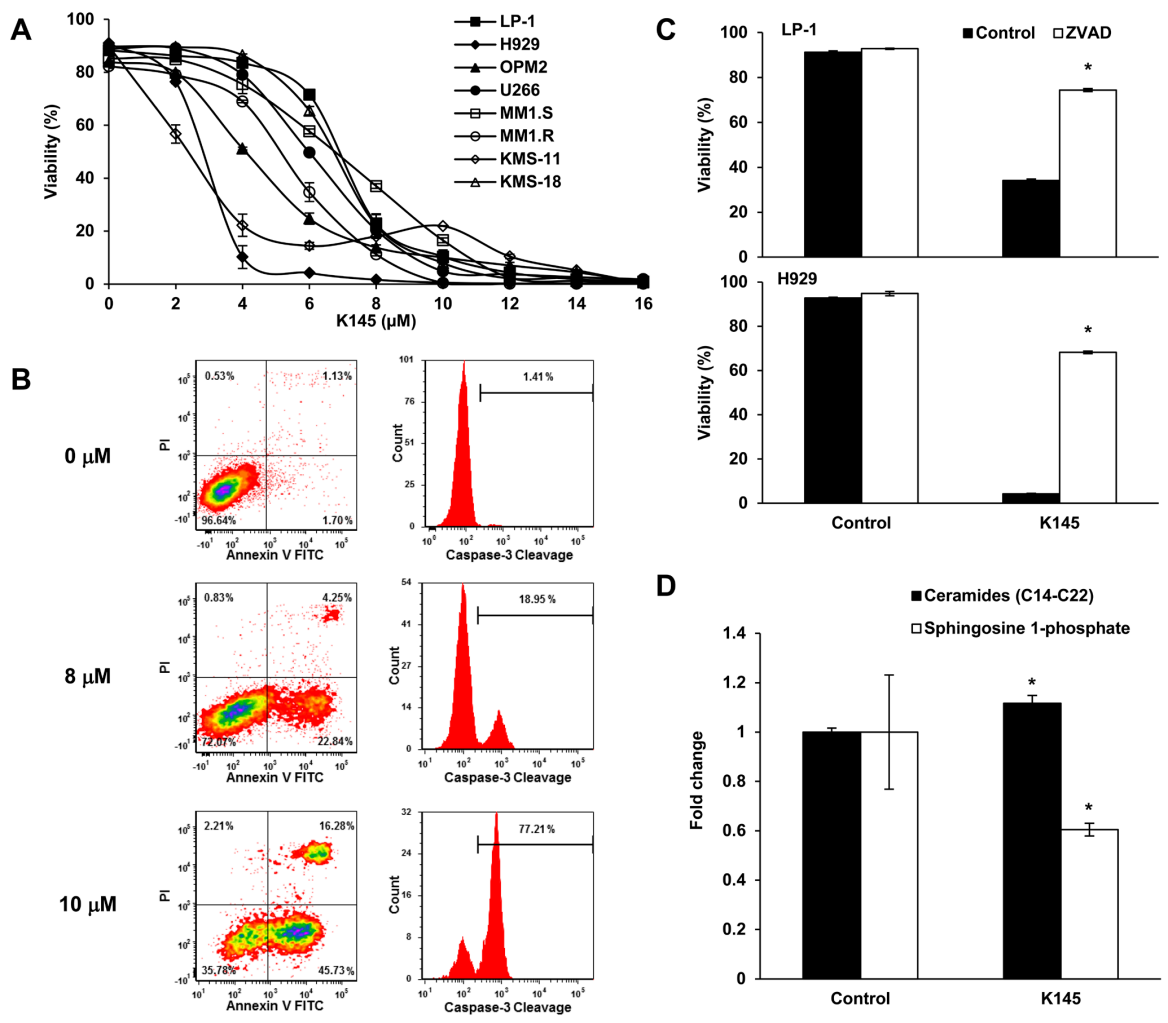
K145 induces myeloma cell death **A**. The indicated human myeloma cell lines were cultured with increasing concentrations of K145 for 24 h and cell viability measured by flow cytometry using Annexin-V and PI staining with dual negative cells considered viable. **B**. LP-1 cells were treated for 16 h with the indicated concentrations of K145 and caspase-3 cleavage determined by intracellular flow cytometry. **C**. LP-1 (upper) and H929 (lower) cells were cultured with 10 μM or 4 μM, respectively, of K145 or vehicle, with or without pre-incubation with 100 μM Z-VAD-FMK and viability assessed after 16 h by flow cytometry. **D**. Sphingolipidomic analysis showing reduced S1P and increased long chain ceramide species in LP-1 cells treated with 8 μM K145 for 6 h. Data are mean±SD of duplicate measurements and are representative of three independent experiments. * *P* < 0.05.

Previous studies examining the targeting of SK2 in myeloma employed ABC294640 [11], a SK2 inhibitor that has been recently shown to display off target effects by more potently inhibiting dihydroceramide desaturase [15, 16]. Thus to validate the effect of inhibiting SK2 in myeloma we treated human myeloma cell lines with a different, more potent SK2-selective inhibitor, K145 [17], which we established does not inhibit dihydroceramide desaturase (Supplementary Figure 2). K145 resulted in myeloma cell death at 24 h with IC_50_ values of 3 to 7 μM as determined by Annexin V/PI staining and flow cytometry (Figure 2A). Consistent with a previous report [11], dose- and time-dependent myeloma cell death was also observed with ABC294640 (Supplementary Figure 3) with IC_50_ values at 24 h being significantly higher than those measured at later timepoints (*P* < 0.001, ANOVA with repeated measures), further suggesting that inhibition of SK2 has anti-myeloma activity. K145-induced cell death was dose-dependent, corresponded to a progressive reduction in cell viability and was associated with caspase-3 cleavage (Figure 2B). This cell death was largely prevented upon pre-incubation of myeloma cells with the pan-caspase inhibitor Z-VAD-FMK prior to the addition of K145, indicative of a caspase-dependent process and classical apoptosis (Figure 2C). As ceramides, upstream of SK2 action, are generally associated with apoptosis [18], we confirmed that K145 was associated with loss of S1P and the accumulation of long-chain (C14-C22) ceramides (Figure 2D and Supplementary Figure 4).

### Blockade of sphingosine kinase 2 synergises with bortezomib to induce myeloma cell death

**Figure 3:**
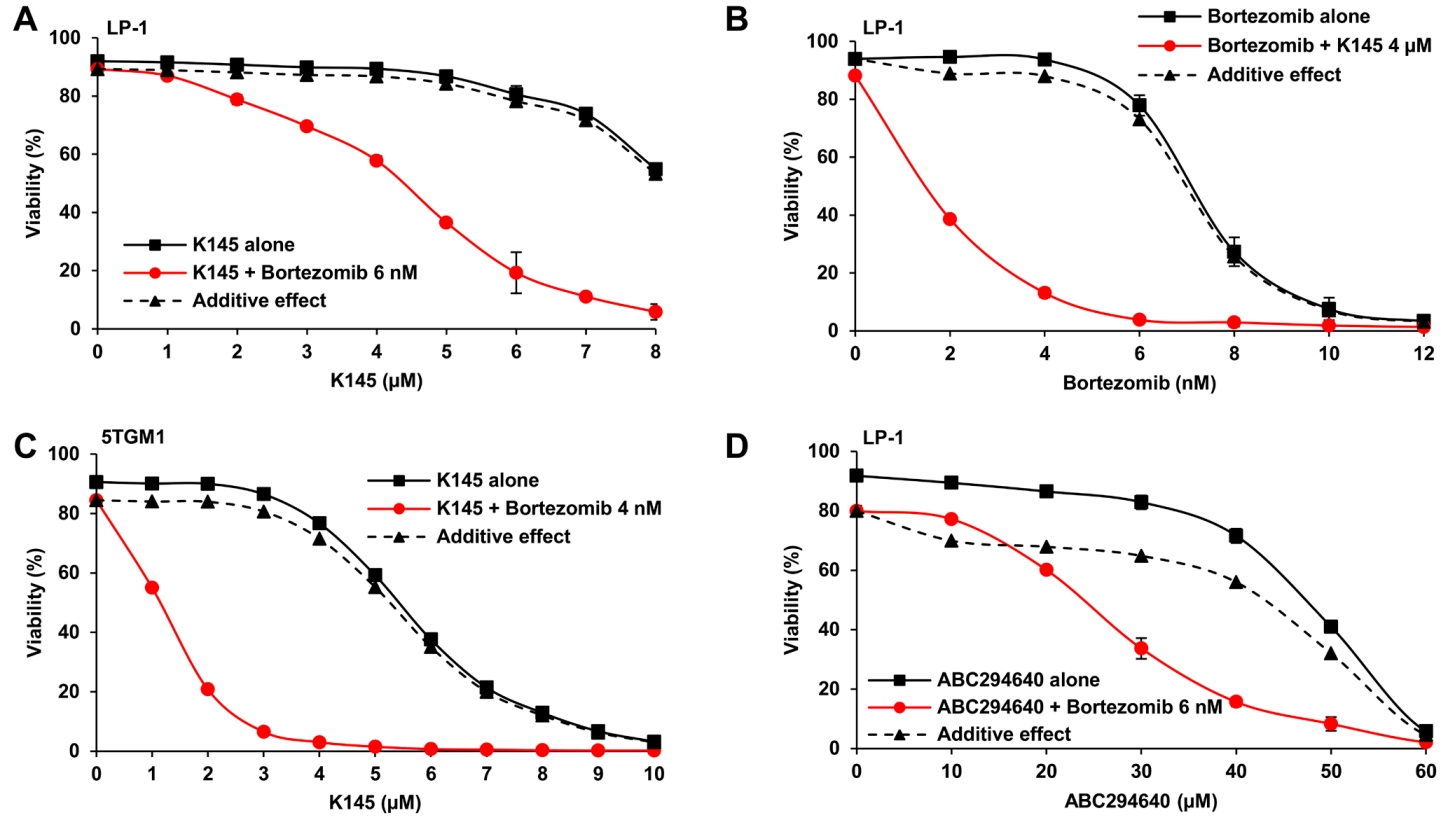
SK2 inhibition synergises with bortezomib **A**. LP-1 cells were cultured with the indicated concentrations of K145 with and without 6 nM bortezomib for 24 h and cell viability measured by flow cytometry using Annexin-V/PI staining. Predicted additive effects, calculated by the fractional product method [19], are shown by the dashed lines whilst actual observed combinational effects by the red lines. **B**. LP-1 cells were cultured with the indicated concentrations of bortezomib with and without 4 μM K145 for 24 h and cell viability measured by flow cytometry using Annexin-V/PI staining. **C**. 5TGM1 cells were cultured with the indicated concentrations of K145 with and without 4 nM bortezomib for 24 h and cell viability measured by flow cytometry using Annexin-V/PI staining. **D**. LP-1 cells were cultured with the indicated concentrations of ABC294640 with and without 6 nM bortezomib for 24 h and cell viability measured by flow cytometry using Annexin-V/PI staining. Predicted additive and observed combinational effects are shown by the dashed and red lines, respectively. Mean±SD of duplicate measurements shown. Data are representative of three independent experiments.

Since the proteasome inhibitor bortezomib is a current front-line therapy for myeloma, we next examined the effect of treating myeloma cell lines with K145 in combination with bortezomib. Strikingly, combinations of minimally cytotoxic concentrations of bortezomib with K145 resulted in strong synergistic cell death in LP-1 (Figure 3A), 5TGM1 (Figure 3C) and H929 (Supplementary Figure 5) human and mouse myeloma cell lines. These synergistic drug interactions were demonstrated by the fractional product method [19] with values less than -0.1 consistent with synergy and were confirmed by application of the combinatorial index (Supplementary Table 1). K145 also synergistically enhanced the efficacy of bortezomib in LP-1 cells, supporting the clinical potential of this combinatorial strategy (Figure 3B and Supplementary Table 2). Notably, combination drug treatment of 5TGM1 cells was associated with caspase-3 cleavage consistent with our observations in human myeloma cells (Supplementary Figure 6A). The alternative SK2 inhibitor, ABC294640, was used to confirm the synergistic effects of targeting SK2 in combination with bortezomib in LP-1 cells. Although relatively high ABC294640 concentrations were required to elicit cell death, a synergistic reduction in cell viability was again observed with bortezomib (Figure 3D and Supplementary Table 1).

**Figure 4:**
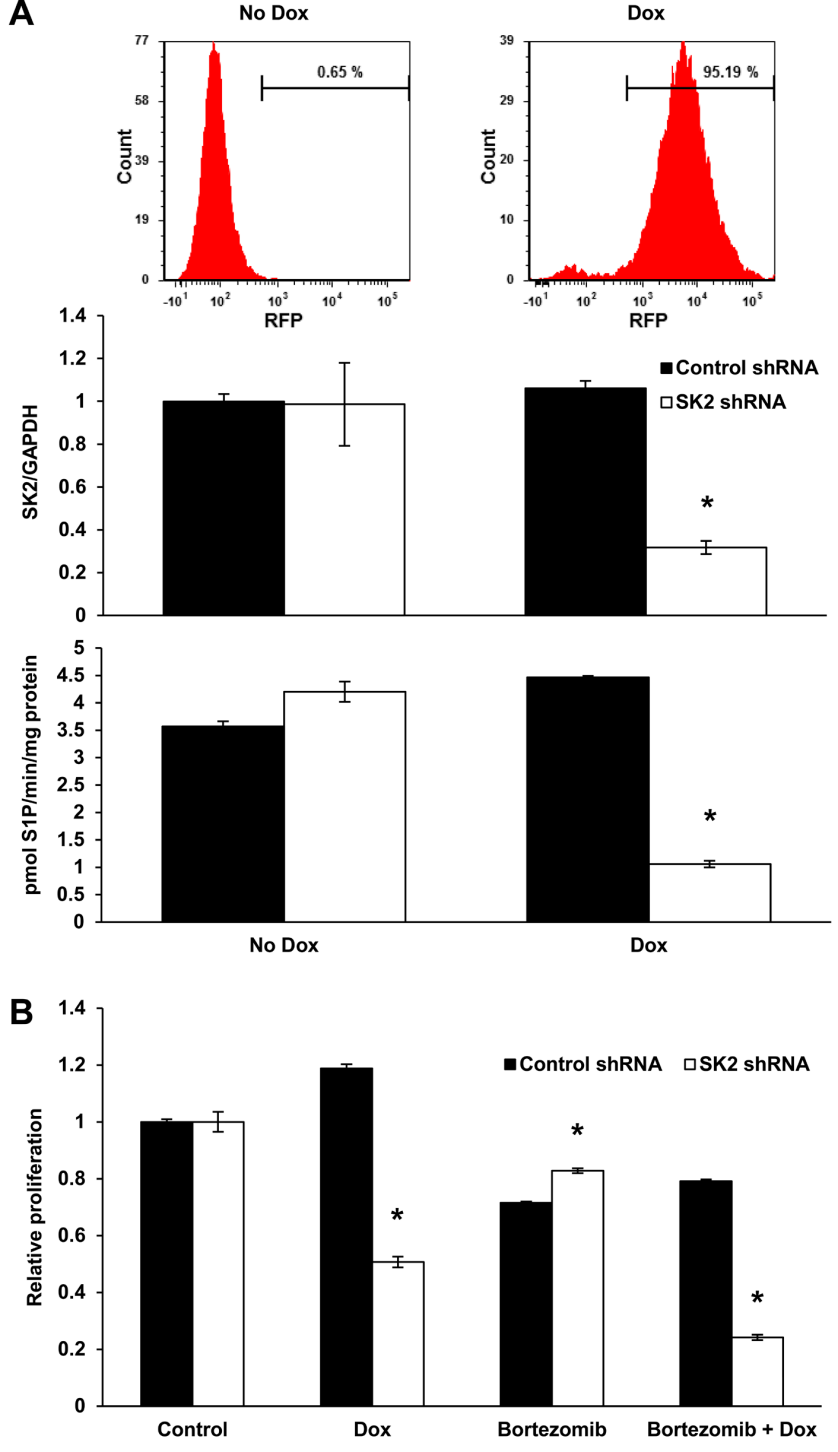
Genetic knockdown of SK2 recapitulates SK2 inhibition using K145 **A**. LP-1 cells were strongly RFP positive after 72 h of culture with 0.5 μg/mL doxycycline (dox) consistent with stable integration of the tetracycline inducible SK2 shRNA into the cell genome. SK2 gene expression and activity were reduced by 65% and 75%, respectively, in LP-1 cells exposed to the indicated concentrations of dox for 72 h. Mean±SD of triplicate measurements are shown. **B**. LP-1 SK2 shRNA inducible cells were treated with 0.5 μg/mL dox for 72 h and 4 nM bortezomib added for the final 24 h of culture. Cell proliferation was measured by WST-1 assay. Reduced SK2 expression and activity results in a 52% reduction in proliferation which was further enhanced by the addition of bortezomib. Mean±SD of quadruplicate measurements shown. Data are representative of at least three independent experiments. * *P* < 0.05.

To confirm that the synergy between K145 and bortezomib was due to inhibition of SK2, we next assessed the effect of SK2 knockdown, using LP-1 cells expressing doxycycline-inducible SK2 shRNA and red fluorescent protein (RFP), in combination with low-dose bortezomib. Following 3 days of doxycycline induction, over 95% of the cells were RFP positive and SK2 knockdown was confirmed by RT-qPCR and SK2 activity assays (Figure 4A). Knockdown of SK2 alone resulted in a reduction in cell proliferation, an effect that was further significantly enhanced by concomitant proteasome inhibition with bortezomib (*P* < 0.001, Figure 4B).

### Bortezomib enhances ER stress induced by sphingosine kinase 2 inhibition and produces a terminal UPR

**Figure 5:**
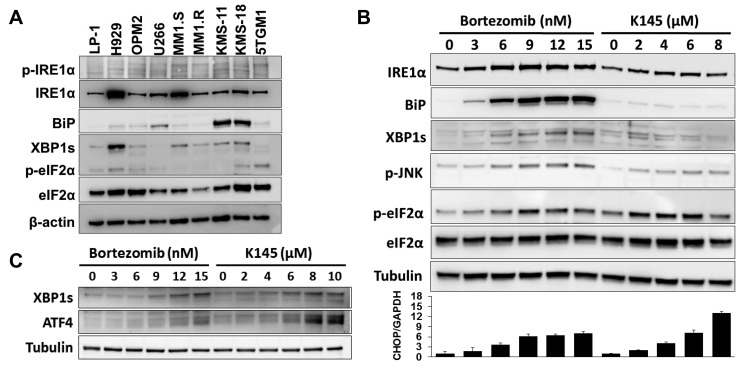
Bortezomib and K145 individually induce ER stress **A**. ER stress and UPR proteins were assessed in a range of human myeloma cell lines and the 5TGM1 murine myeloma cell line. β-actin was used as a loading control. **B**. LP-1 cells were exposed to increasing concentrations of either bortezomib or K145 for 16 h and examined by Western blot for ER stress and UPR proteins. α-tubulin was used as a loading control. The viabilities for bortezomib conditions were 92%, 90%, 86%, 67%, 58% and 51%, whilst those for K145 were 91%, 88%, 84%, 78% and 68%, respectively. RT-qPCR analysis for CHOP was performed in parallel and presented as a bar chart below the Western blots. Mean±SD of triplicate measurements shown. Data are representative of three independent experiments. **C**. LP-1 cells were exposed to increasing concentrations of either bortezomib or K145 for 4 h and examined by Western blot for ER stress and UPR proteins. α-tubulin was used as a loading control. The viabilities for all conditions were greater than 85%.

Myeloma cells are known to have elevated basal ER stress and activation of the UPR [4]. Analysis of a panel of human myeloma cell lines for several markers of ER stress and the UPR, including the ER chaperone BiP, XBP1s and phosphorylated eIF2α (p-eIF2α), showed marked variation in their basal expression (Figure 5A), implying differential sensitivities to proteasome inhibitors between myeloma cells. The ATF6 protein was not detectable in three of the human myeloma cell lines tested (LP-1, H929 and OPM2) and was not analysed further.

By inhibiting the proteasome, bortezomib has been shown to induce sufficient ER stress to activate pro-apoptotic UPR signalling [7]. ER stress-induced activation of the endoribonuclease domain of the ER transmembrane protein, IRE1, splices an intron from precursor XBP1 mRNA (XBP1u) that results in translation of the active transcription factor XBP1s [20, 21], levels of which have been shown to correlate with sensitivity to bortezomib in myeloma patients [22]. While bortezomib induces classical ER stress [7], the ER stress-inducing potential of SK2 inhibition was unknown. Notably, however, SK2 can localise to the ER [9, 10], and ceramide, a major pro-apoptotic lipid upstream of sphingosine kinase action has been widely implicated in inducing ER stress, mostly in non-cancer settings [23–25]. To examine the levels of ER stress and UPR activation in response to either bortezomib or K145 alone, LP-1 cells were exposed to increasing concentrations of either drug and assessed for several ER stress and UPR markers (Figure 5B). Consistent with previous reports, bortezomib induced a classical ER stress signalling profile with dose-dependent increases in BiP, XBP1s and p-eIF2α observed at relatively long timepoints (16 hours). SK2 inhibition by K145 also induced ER stress as indicated by dose-dependent increases in XBP1s and p-eIF2α. However, in contrast to bortezomib, BiP increased to a lesser degree in response to K145 (Figure 5B), a finding also seen in a second human myeloma cell line, OPM2 and the murine myeloma cell line, 5TGM1 (Supplementary Figures 6B, 7A and 7B). Consistent with prolonged ER stress, both bortezomib and K145 induced expression of the transcription factor CHOP downstream of p-eIF2α (Figure 5B). Interestingly, increased expression of IRE1α and modest activation of the apoptosis inducing stress kinase JNK were also observed with both agents (Figure 5B and Supplementary Figure 7C). Since induction of ER stress is likely an early event in response to these drugs, similar experiments performed at the early time point of 4 hours, prior to measurable cell death, demonstrated expression of ATF4 downstream of p-eIF2α and XBP1s with both bortezomib and K145 (Figure 5C and Supplementary Figure 7A).

**Figure 6:**
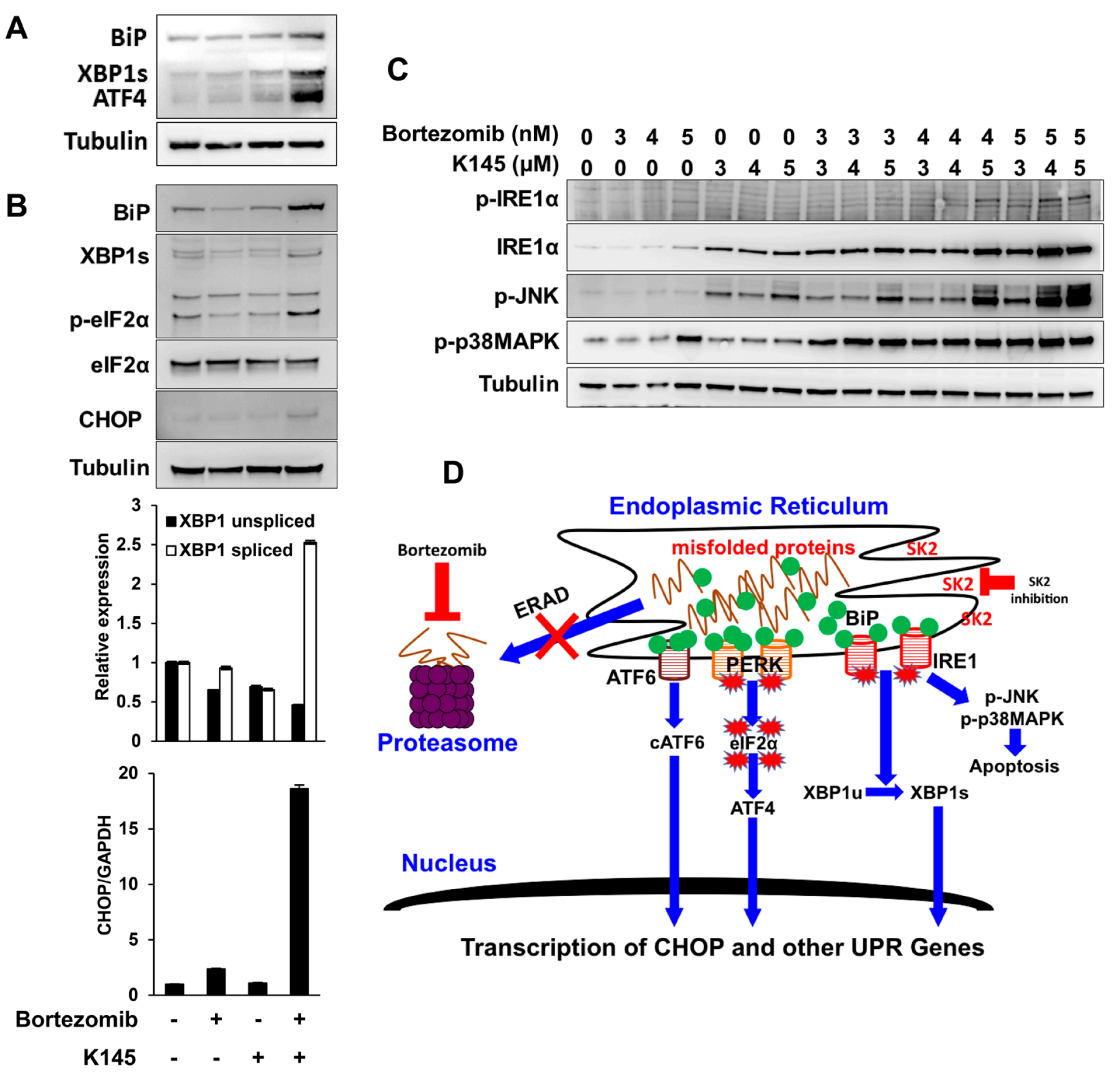
Dual bortezomib and K145 treatment induces synergistic ER stress **A**. LP-1 cells were cultured with vehicle and sub-cytotoxic concentrations of bortezomib (5 nM), K145 (7 μM) or both for 4 h and examined for levels of ER stress and UPR activation by Western blot. **B**. LP-1 cells were cultured with vehicle and sub-cytotoxic concentrations of bortezomib (3 nM), K145 (4 μM) or both for 16 h and examined for levels of ER stress and UPR activation by Western blot. Co-administration of bortezomib and K145 to LP-1 cells increases expression of spliced XBP1 mRNA and produces a marked increase in CHOP gene expression by RT-qPCR at 16 h shown as bar charts below the Western blots. Mean±SD of triplicate determinations are shown with expression levels relative to control cultures and normalised to GAPDH. **C**. LP-1 cells were cultured with the indicated concentrations of bortezomib and K145 for 16 h. Levels of IRE1α and stress kinase activation were assessed by Western blot. α-tubulin was used as a loading control. **D**. Proposed schematic of how bortezomib and K145 combine to induce synergistic ER stress and UPR activation that results in pro-apoptotic IRE1α signalling.

In view of the observation that like bortezomib, the SK2 inhibitor K145 induced ER stress, we next assessed the potential for K145 to synergise with bortezomib to enhance ER stress in myeloma cells. The combination of individually sub-cytotoxic concentrations of bortezomib and K145 resulted in early synergistic expression of BiP, ATF4 and XBP1s (Figure 6A) with synergistic increases in BiP, XBP1s, p-eIF2α and CHOP detectable at later time points, consistent with strong induction of ER stress and UPR activation (Figure 6B). Similar findings were obtained using OPM2 and 5TGM1 cells (Supplementary Figures 6C, 7A and 7B), although in these cell lines, dephosphorylation of eIF2α was observed with the combination of both agents at longer culture times which occurs in the setting of chronic, unrecoverable ER stress and subsequent initiation of cell death [26]. However ATF4, which is downstream of p-eIF2α, was increased in OPM2 cells, clearly demonstrating activation of this pathway (Supplementary Figure 7A). As XBP1s protein is expressed early after the initiation of ER stress and is degraded rapidly in response to its chronicity [27], PCR was used to overcome difficulties in detecting XBP1s at later time points. RT-qPCR revealed a modest decrease in XBP1u and no significant increase in XBP1s following 16 h treatment with either bortezomib or K145 alone (Figure 6B). However, the combination of the two drugs resulted in a 2.5-fold increase in XBP1s. Additionally, a 2.4-fold increase in CHOP expression was observed at 16 h with the sub-cytotoxic concentration of bortezomib, whilst the sub-cytotoxic concentration of K145 did not significantly alter expression, as expected (Figure 6B). Consistent with the synergistic cell death seen when combining K145 with bortezomib, a synergistic 19-fold increase in CHOP expression was detected at 16 h (Figure 6B). Furthermore, the combination of bortezomib with K145 at various sub- or minimally cytotoxic concentrations resulted in a synergistic increase in IRE1α expression, which was associated with activation of the stress kinases JNK and p38MAPK (Figure 6C and Supplementary Figure 7C). Similar synergistic induction of ER stress, as shown by increases in XBP1s and ATF4, was also observed with sub-cytotoxic doses of bortezomib in combination with the alternate SK2 inhibitor, ABC294640 (Supplementary Figure 8). Taken together, these data suggest that combining proteasome and SK2 inhibition results in synergistic ER stress and a terminal UPR that is associated with activation of stress kinases and subsequent apoptosis (Figure 6D).

### K145 synergises with bortezomib in an aggressive murine myeloma model

Since synergistic cell death was also observed in 5TGM1 murine myeloma cells treated with bortezomib and K145 (Figure 3C), we next examined the effects of combinations of bortezomib and K145 *in vivo*. The 5TGM1 C57BL/KaLwRij murine model of myeloma recapitulates aggressive human myeloma in an immunocompetent setting, with the rapid development of bone marrow failure and lytic bone lesions necessitating a relatively short (4-week) experimental design [28–34]. C57BL/KaLwRij mice were injected with 1 million 5TGM1 cells and disease was allowed to develop for two weeks before administration of bortezomib and/or K145. Disease burden was assessed by bioluminescence imaging after luciferin injection at baseline (day 14), after one week of treatment (day 21) and after two weeks of treatment (day 28) (Supplementary Figure 9). The 0.5 mg/kg dose of bortezomib was chosen as it approximates the 1.3 mg/m^2^ dose administered to patients [35]. In a pilot study, K145 was administered daily at 40 mg/kg daily which, while resulting in significant reductions in disease burden compared to vehicle treated controls, mice exhibited signs of toxicity including lethargy, reduced food intake and weight loss. Thus, doses of K145 were reduced to 20 mg/kg in order to both reduce toxicity, and also to have minimal anti-myeloma effect so that the question of *in vivo* synergy with bortezomib could be evaluated.

**Figure 7:**
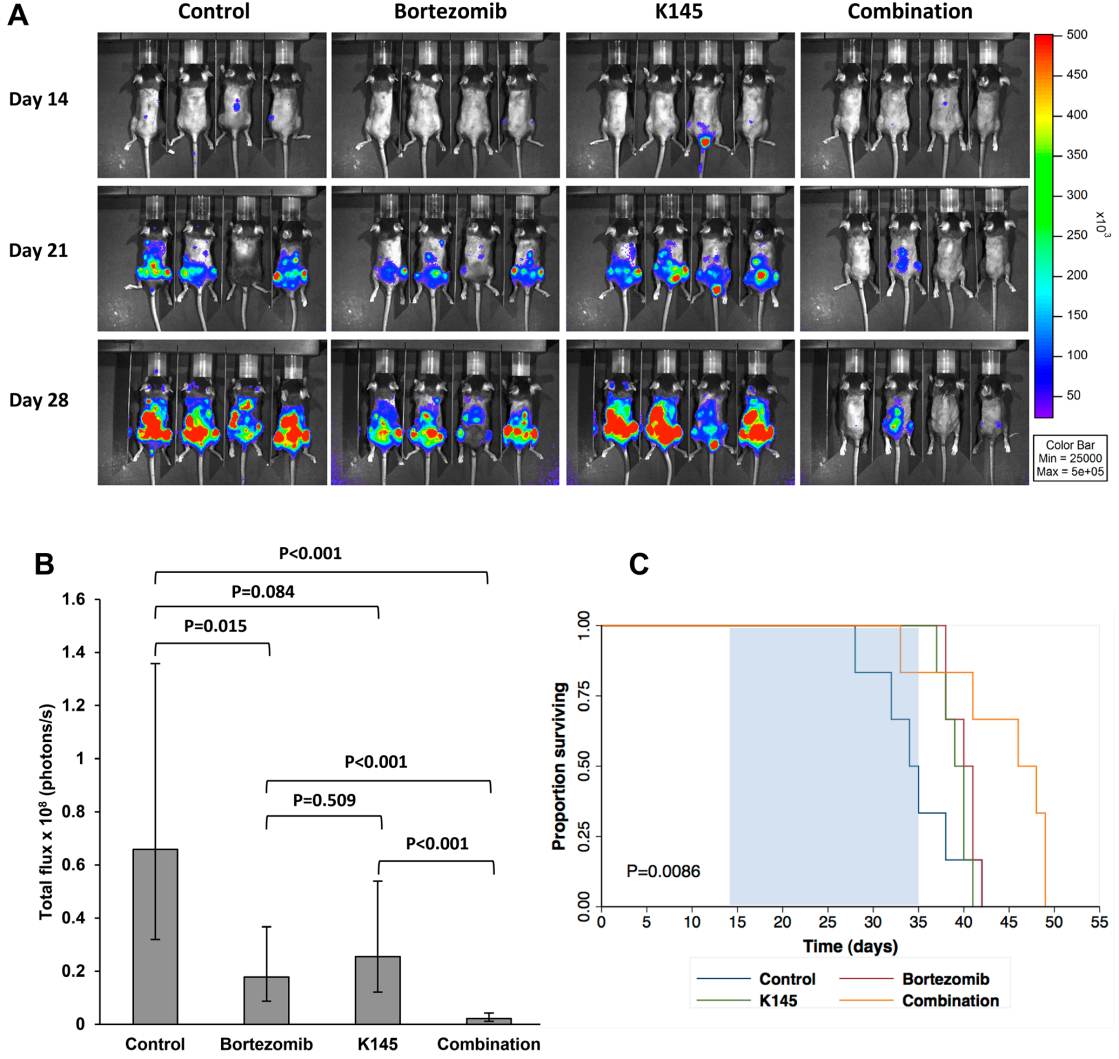
Dual bortezomib and K145 therapy shows *in vivo* efficacy in the aggressive C57BL/KaLwRij murine myeloma model **A**. Representative bioluminescence images of mice in each treatment group after luciferin injection at day 14 (pre-treatment), day 21 (after one week of treatment) and day 28 (after two weeks of treatment). **B**. Quantitation of myeloma disease burden in each treatment group at day 28. Disease burden at each timepoint was modelled using a multiple linear regression that takes into consideration varying baseline (day 14) disease levels between animals. Mean disease burden ± 95% confidence interval for the mean are shown. 13 (7 male, 6 female), 13 (7 male, 6 female), 13 (7 male, 6 female) and 15 (9 male, 6 female) mice were analysed from the control, bortezomib, K145 and combination treatment groups, respectively. **C**. Mice were treated for three weeks (days 14 to 35) indicated by the shaded region and then monitored for clinical deterioration and culled as required. Kaplan-Meier survival functions for each treatment group are shown (*P* = 0.0085, log-rank test).

Administration of 0.5 mg/kg bortezomib as a monotherapy, three times per week for two weeks, resulted in a modest reduction in myeloma burden, consistent with previous reports [29, 30, 35]. Daily administration of 20 mg/kg K145 as a monotherapy, for two weeks, also gave a modest reduction in myeloma burden, supporting the findings of a previous study using an alternative, poorly selective SK2 inhibitor, ABC294640 [11]. The combination of bortezomib and K145, however, resulted in a remarkable synergistic reduction in myeloma burden (Figure 7A and 7B), with vehicle control mice possessing 31 times more disease than mice receiving both drugs (*P* < 0.001, 95% CI 10.82-87.61). Mice receiving single drug therapy did not lose weight, nor manifest any signs of drug toxicity, compared to vehicle controls whilst a small, non-significant reduction in weight was observed in mice receiving both drugs (mean weight reduction 1.8±0.5 g, *P* = 0.192). Furthermore, when treatment was extended for an additional week to where clinical deterioration enabled a survival profile to be obtained, mice receiving both bortezomib and K145 showed a median 12-day extension in survival (median survival 46 days, 95% CI 33 days to 58 days) compared to vehicle control mice (median survival 34 days, 95% CI 28 days to 39 days) (Figure 7C).

### Dual K145 and bortezomib therapy reduces myeloma-associated bone disease

**Figure 8:**
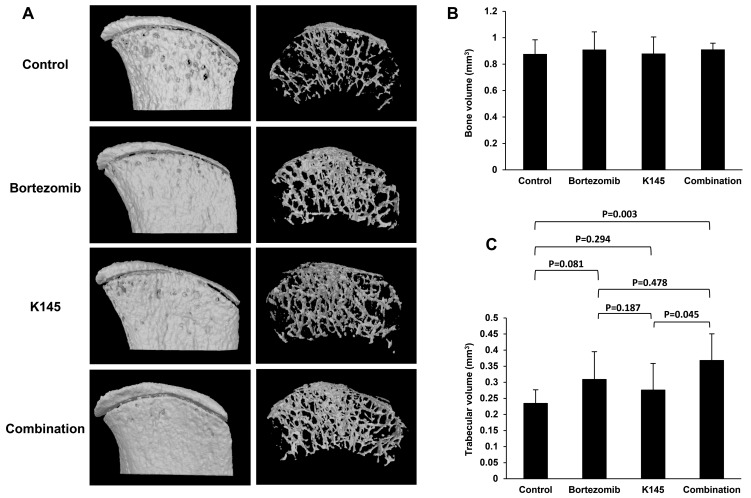
Dual bortezomib and K145 therapy has favourable effects on myeloma bone disease **A**. Representative micro CT scans of right-sided iliac crests from female mice in each treatment group. External bone surface (left) is shown to visualise lytic lesions whilst the interior of the bone (right) enables assessment of trabecular integrity. **B**. Quantitation of total bone volume between groups. **C**. Quantitation of trabecular volume between treatment groups. Mice receiving the combination of bortezomib and K145 had an average total trabecular volume of 0.37±0.08 mm^3^ *versus* 0.23±0.04 mm^3^ in vehicle-treated animals (*P* = 0.003) and 0.28±0.08 mm^3^ in K145-treated animals (*P* = 0.045). However, there was only a trend towards greater trabecular volume compared to bortezomib treated mice, 0.37±0.08 mm^3^ *versus* 0.31±0.09 mm^3^ in bortezomib-treated animals (*P* = 0.478). Six female mice were analysed per group with mean±SD shown.

Myeloma results in the dysregulation of bone remodelling in favour of increased osteoclastic resorption resulting in many of the deleterious symptoms, including generalised bone loss, lytic bone lesions, hypercalcaemia and pathological fracture [1]. As bortezomib is known to reduce the number and severity of bone lytic lesions [36–38], we hypothesized that the addition of K145 to bortezomib would result in a further reduction in lytic lesions compared to either drug alone or vehicle. In mice receiving both drugs, micro CT analysis of the iliac crests, a region frequently infiltrated with myeloma according to bioluminescence images, showed reduced lytic lesions and greater trabecular integrity compared with other groups (Figure 8A and Supplementary Figure 10). However, no observable changes in total bone volume between treatment groups were evident (Figure 8B). Further analysis of trabecular volume demonstrated that mice receiving the combination of bortezomib and K145 had significantly greater total trabecular volume compared to vehicle control mice or mice treated with K145 alone (Figure 8C). A similar trend towards greater trabecular volume was also observed in combination treated mice compared to those treated with bortezomib alone, although this did not reach statistical significance (Figure 8C).

## DISCUSSION

To date, most of the studies examining the role of the sphingosine kinases in cancer have focused on SK1 [39, 40]. However, studies have highlighted an emerging role for SK2 in haematological malignancies, including ALL [9–11]. A recent study by Venkata and colleagues [11] revealed a role of SK2 in myeloma disease pathogenesis and highlights SK2 as a potential therapeutic target. The subcellular localisation of SK2 to intracellular compartments, such as the ER and nucleus, suggest highly specialised roles for which ER membrane integrity and cell cycle regulation through S1P-induced inhibition of histone deacetylases have been reported [10, 41, 42]. In this study, we examined the relevance of SK2 in myeloma and show that inhibition of SK2 function can synergise with the clinically important proteasome inhibitor, bortezomib, to induce enhanced ER stress and UPR activation and attenuate myeloma cell growth and survival.

Perturbation of lipid metabolism, including the accumulation of ceramide species has been shown to induce ER stress and activate the UPR [23–25]. The *de novo* ceramide synthesis pathway occurs at the ER membrane so manipulation of this pathway can alter ER membrane sphingolipid homeostasis and contributes to ER stress [43–45]. However, these findings highlight the potential for a different mechanism of ER stress induction from that seen with proteasome inhibitors which result in the accumulation of misfolded proteins in the ER lumen, BiP dissociation from the lumenal domains of PERK, ATF6 and IRE1, chaperoning of misfolded proteins and activation of the UPR [7]. In keeping with this hypothesis, it has recently been shown that changes in the lipid composition of the ER membrane can directly activate IRE1 and PERK independently of lumenal unfolded proteins, to induce the UPR in a process that requires their transmembrane domains [46]. Our data show that K145 inhibition of SK2 results in increased ceramide species that, consistent with an ER membrane effect, does not significantly induce BiP expression but activates the PERK-eIF2α and IRE1-XBP1 pathways (Figure 5). This is in contrast to bortezomib, which results in strong BiP expression consistent with initiation of ER stress within the ER lumen. These different mechanisms leading to ER stress induced by bortezomib and K145 provide considerable rationale for concomitantly targeting both pathways in myeloma.

When used as single agents, or in combination, K145 and bortezomib induce JNK and p38MAPK activation, both components of well-established pro-apoptotic signalling cascades. It has been shown that activation of the kinase domain of IRE1 is required for its endoribonuclease activity to splice XBP1 mRNA [21], however, IRE1 kinase activity also stimulates activation of tumour necrosis factor receptor associated factor 2 (TRAF2) and apoptotic-signalling kinase-1 (ASK1) which, in turn, activate JNK and p38MAPK to induce apoptosis. [47] We have found that the IRE1-JNK pathway correlates with myeloma cell death induced by bortezomib or K145 and is markedly enhanced when both agents are used in combination. CHOP expression also increased markedly upon combining bortezomib with K145 treatment. This observation, combined with the previous studies demonstrating that p38MAPK can activate CHOP *via* phosphorylation of its transactivation domain [48], suggests the potential for ER stress in myeloma cells to connect to the cellular apoptotic machinery through both the IRE1-JNK/p38MAPK pathway and PERK-eIF2α-CHOP signalling, particularly in the context of SK2 inhibition.

Combination therapy with bortezomib and K145 was highly effective in the aggressive C57BL/KaLwRij 5TGM1 murine myeloma model. Either drug alone produced only modest anti-myeloma effects after two weeks of treatment. However, the synergistic reduction in disease burden between animals receiving both drugs compared to single drug or vehicle was striking and suggests clinically relevant therapeutic potential for combination therapy with both bortezomib and an inhibitor of SK2. Furthermore, the reduction in lytic bone lesions and preservation of trabecular volume support the biological activity of this combination drug therapy, though these findings warrant further detailed studies. Whether these effects are at least, in part, related to bone marrow microenvironmental changes are yet to be examined.

Notably, our combination of K145 with bortezomib did not result in any obvious toxicity in mice. Furthermore, ongoing clinical trials with SK2 inhibitors have not reported any drug-related toxicities in humans [11], including neurotoxicity which is a common side-effect of bortezomib [49]. Indeed, in some contexts SK2 inhibition appears neuroprotective [50]. Thus, combining SK2 inhibitors with bortezomib appears feasible in humans.

In summary, this study confirms that SK2 expression and activity are elevated compared to SK1 in myeloma cells and provides pre-clinical evidence for incorporating an inhibitor of SK2 into a proteasome inhibitor-containing combination drug therapeutic regimen. The tight control of ER stress in the myeloma cell also represents a potential weakness in its armoury, which is exploited by proteasome inhibitors and can be further enhanced by SK2 inhibition. Mechanistically, the importance of membrane lipids in contributing to ER stress levels and inducing UPR activation in myeloma cells has not been examined and represents a promising avenue of exploration that, in a biologically plausible manner, synergistically contributes to lumenal factors that initiate ER stress. More potent SK2 inhibitors and modulators of sphingolipid signalling are currently in development and are expected to further highlight the unappreciated role of sphingolipid biology in myeloma, which will ultimately lead to future therapeutic options.

## MATERIALS AND METHODS

### Cell lines

The human myeloma cell lines LP-1, NCI-H929, OPM2, U266, MM1.S, MM1.R, KMS-11, KMS-18, were grown in RPMI with 10% foetal calf serum, 100 U penicillin, 0.1 mg/mL streptomycin, 2 mM L-glutamine, 1 mM pyruvate and 15 mM Hepes buffer in a humidified 5% CO_2_ atmosphere at 37°C. The 5TGM1 murine myeloma cell line, which constitutively expresses luciferase and GFP [31], was grown in IMDM with 20% foetal calf serum and otherwise identical conditions to the human myeloma cell lines.

### Reagents, antibodies and western blotting

The SK2 inhibitors, K145 [17] and ABC294640 [51], were purchased from Medkoo Biosciences (Chapel Hill, NC). Bortezomib (Janssen Cilag, New Brunswick NJ) was kindly provided by the Royal Adelaide Hospital Pharmacy Department. Luciferin was purchased from Biosynth (Lake Constance, Switzerland). Details of other reagents, antibodies and Western blotting are provided in Supplementary Methods.

### Sphingolipidomics

Quantitation of sphingolipid species was performed at the Lipidomics Core Facility, Medical University of South Carolina, using previously described methods [52].

### Proliferation and viability assays

Cell proliferation was measured using the WST-1 reagent (Sigma-Aldrich). Briefly, 1 × 10^4^ myeloma cells were plated in 100 μL culture medium (control) or varying concentrations of drug for 72 h after which 10 μL of WST-1 reagent was added to each well for up to 2 h and absorbance measured at 450 nM using a BiTek Epoch Microplate Spectrophotometer (Winooski, VT). Cell viability was measured using Annexin-V FITC and either PI or 7AAD staining as previously described [9] and analysed using a LSRFortessa flow cytometer (BD Biosciences).

### Lentiviral SK2 shRNA

Details of the generation and transduction of the lentivirus encoding short hairpin RNA (shRNA) targeting SK2 are described in the Supplementary Methods.

### Quantitative RT-PCR

Details of quantitative RT-PCR are provided in the Supplementary Methods.

### *In vivo* myeloma model

Six to eight-week old C57BL/KaLwRij mice were inoculated with 1 × 10^6^ 5TGM1 cells by tail vein injection and disease allowed to develop for two weeks. Mice were then divided into four treatment groups: vehicle control, bortezomib alone, K145 alone and the combination of bortezomib and K145. K145 was administered daily at 20 mg/kg whilst bortezomib was administered three times per week at 0.5 mg/kg. Both drugs were given by intraperitoneal (IP) injection. Bioluminescence imaging after IP injection of 100 μL 30 mg/mL luciferin was performed at day 14 (prior to treatment), day 21 (after 1 week of treatment) and day 28 (after two weeks of treatment). Mice were used with permission from the SA Pathology/Central Adelaide Local Health Network Animal Ethics committee (approval number 08a/14) and experiments were performed under the guidelines from the Australian code for the care and use of animals for scientific purposes, 8^th^ edition, 2013. Details of micro CT analysis are provided in the Supplementary Methods.

### Statistical analysis

Details of statistical analysis are provided in the Supplementary Methods.

## SUPPLEMENTARY MATERIALS FIGURES AND TABLES



## Abbreviations

MGUS: monoclonal gammopathy of undetermined significance
ER: endoplasmic reticulum
UPR: unfolded protein response
SK1: sphingosine kinase 1
SK2: sphingosine kinase 2
S1P: sphingosine 1-phosphate
ERAD: ER-associated degradation
PERK: protein kinase R-like ER kinase
IRE1: inositol-requiring kinase 1
ATF6: activating transcription factor 6
eIF2α: eukaryotic translation initiation factor 2α
ATF4: activating transcription factor 4
XBP1: x-box binding protein 1
CHOP: CCAAT/enhancer binding protein (C/EBP) homologous protein
BiP: binding immunoglobulin protein
JNK: c-Jun N-terminal kinase
p38MAPK: p38 mitogen-activated protein kinase
TRAF2: tumour necrosis factor receptor associated factor 2
ASK1: apoptotic-signalling kinase-1 (ASK1)
RFP: red fluorescent protein.
