# Supplementary material for: Sphingosine kinase 2 inhibition synergises with bortezomib to target myeloma by enhancing endoplasmic reticulum stress
Source: Oncotarget. 2017 Apr 14;8(27):43602–16. doi: 10.18632/oncotarget.17115 (PMC5546428; doi:10.18632/oncotarget.17115)
Supplement: Supplementary file 1 [file oncotarget-08-43602-s001.pdf]

Sphingosine kinase 2 inhibition synergises with bortezomib to target myeloma by enhancing endoplasmic reticulum stress

Supplementary Material

METHODS

Lentiviral SK2 shRNA constructs

pTRIPZ (Open Biosystems, Lafayette CO) was modified with the addition of a novel polylinker following digestion with ClaI/MluI and ligation of annealed oligonucleotides 5'-CGATGAATTCGTTAACCTCGAGA-3' and 5'-CGCGTCTCGAGGTTAACGAATTCAT-3'. Primers 5'-TAGAATTCTCGACTTCTTAACCCAACAGAAGGCTCGAGAAGGTATATTGCTGTTGACAGTGAGCG-3' and 5'-TCTCGAATTCTAGCCCCTTGAAGTCCGAGGCAGTAGGC-3' and the following 97-mer oligonucleotide templates [4] were used to amplify the mir30/shRNA target sequences:

Ren713 5'-TGCTGTTGACAGTGAGCG CAGGAATTATAATGCTTATCTATAGTG AAGCCACAGATGTATAGATAAGCATTATAATTCCTATGCCTACTGCCTCGGA-3'

SK2(1) 5'-TGCTGTTGACAGTGAGCGACCGGTTGCTTCTATTGGTCAATAGTG AAGCCACAGATGTATTGACCAATAGAAGCAACCGGGTGCCTACTGCCTCGGA-3'

SK2(2) 5'-TGCTGTTGACAGTGAGCGACGGCGCTAGGATTTGCACTAATAGTG AAGCCACAGATGTATTAGTGCAAATCCTAGCGCCGGTGCCTACTGCCTCGGA-3'

SK2(3) 5'-TGCTGTTGACAGTGAGCGAAGGGTAGTGCCTGATCAATGATAGTG AAGCCACAGATGTATCATTGATCAGGCACTACCCTCTGCCTACTGCCTCGGA-3'.

PCR products were digested with EcoRI and cloned into pTRIPZ(PL) to produce shRNA constructs pTRIPZ-Ren713 (negative control), pTRIPZ-SK2(1), pTRIPZ-SK2(2) and pTRIPZ-SK2(3) respectively. A triple shRNA containing vector, pTRIPZ-SK2(1-3), was then generated using blunt ClaI and MluI cassettes and ligated sequentially into pTRIPZ-SK2(1) and then pTRIPZ-SK2(1,2) following digestion with HpaI and MluI. Sequencing verified integrity and orientation of all the respective shRNA vectors.

Reagents and antibodies

The SK2 inhibitors, K145 [5] and ABC294640 [6], were purchased from Medkoo Biosciences (Chapel Hill, NC). Bortezomib (Janssen Cilag, New Brunswick NJ) was kindly provided by the Royal Adelaide Hospital Pharmacy Department. Luciferin was purchased from Biosynth (Lake Constance, Switzerland). The following antibodies were purchased from Cell Signalling Technology (Berverly, MA): IRE1α, BiP, XBP1s, eIF2α, p-eIF2α (Ser51), CHOP, p-JNK (Thr183/Tyr185) and p-p38MAPK (Thr180/Tyr182). Antibodies to p-IRE1α (ser724) were purchased from Novus Biologicals (Littleton, CO). Antibodies to α-tubulin and β-actin were purchased from Abcam (Cambridge, MA). The following reagents were purchased from BD Biosciences (Franklin Lakes, NJ): Annexin-V FITC, 7-Aminoactinomycin (7AAD), anti-cleaved Caspase-3 FITC and Z-VAD-FMK. Propidium iodide (PI) was purchased from Sigma-Aldrich (St Louis, MO).

Western Blotting

Protein lysates of myeloma cell lines were obtained as previously described [2] and loaded into Criterion precast polyacrylamide gels (Bio-Rad, Hercules CA) prior to transferring onto nitrocellulose membranes. Membranes were incubated overnight with primary antibodies in 5% bovine serum albumen or 5% low-fat milk prior to incubation with HRP-conjugated secondary antibodies at room temperature for 2 to 3 h. Proteins were detected after the addition of Clarity ECL Substrate (Bio-Rad) and chemiluminescence detected using the LAS-4000 imaging system (GE Healthcare Life Sciences, Little Chalfont UK). α-tubulin or β-actin were used as loading controls.

Generation of stably expressing tetracycline-inducible SK2 shRNA myeloma cell lines

Replication incompetent lentiviral particles were obtained after transient transfection of HEK293T cells using lipofectamine 2000 (Life Technologies, Carlsbad CA). Lentiviral supernatant was used to transduce human myeloma cell lines in the presence of 4 µg/mL polybrene for 72 h after which transduced cells were selected by the addition of 1 µg/mL puromycin to cultures.

Quantitative RT-PCR

RNA was extracted using the Qiagen RNEasy Mini Kit (Hilden, Germany) and cDNA obtained using the Qiagen Quantitect cDNA Synthesis Kit. Quantitative RT-PCR was performed on a Corbett Rotor-Gene 6000 thermal cycler (Corbett Life Science, Australia) using SYBR Green Real-time PCR Master Mix (Invitrogen, Carlsbad, CA) with thermal cycling conditions: 50°C for 2', 95°C for 2', 40 cycles of 95°C for 15'', 60°C for 15'' and a final melt of 55-95°C. Products were normalized to glyceraldehyde-3-phosphate dehydrogenase (GAPDH) and analyzed on Rotor-Gene software using either quantitative or comparative quantitative analysis with the latter normalized to untreated control samples. The following primers were used: SK1 5'-TCTGGTGGTCATGTCTGGAG-3' and 5'-GACCTGCTCATAGCCAGCAT-3'; SK2 5'-TGGCAGTGGTGTAAGAACCA-3' and 5'-CAGTCAGGGCGATCTAGGAG-3'; XBP1u 5'-GGAGTTAAGACAGCG-3' and 5'-GTCAATACCGCCAGA-3'; XBP1s 5'-TGCTGAGTCCGCAGC-3' and 5'-GCTGGCAGGCTCTGG-3'; CHOP 5'-CAGAGCTGGAACCTGAGGAG-3' and 5'-TGGATCAGTCTGGAAAAGCA-3'; GAPDH 5'-AGCCACATCGCTCAGACAC-3' and 5'-GCCCAATACGACCAAATCC-3'.

Micro CT analysis

Computed tomography images were obtained using a SkyScan-1076 *in vivo* µCT scanner (Bruker MicroCT, Kontich Belgium) for a total of 18 mice preserved in formalin for this study. To remove soft X-rays, a 0.5 mm thick aluminium filter was placed in front of the X-ray source and the images were acquired at 48 kV and 110 µA with a scan resolution of 8.65 µm/pixel. At each angular rotation step of 0.5°, 1 image was acquired. Reference images without specimens were taken before each acquisition for flat field correction. Two-dimensional radiographic projection images were acquired as TIF files and reconstructed into BMP files using a cone-beam algorithm (NRecon Recontruction software, Bruker), with a beam hardening setting of 30% and ring artefact reduction of 10. The reconstructed BMP datasets were opened in Dataviewer (Bruker) and rotated in *x*-, *y*- and *z*- directions, to ensure that the volume of interest (VOI) to be created was in the correct position and angle. A new realigned dataset was then generated for each VOI and imported into CTAn software (Bruker). The top of the pelvis was identified and 1581 slices were selected and saved as a new VOI for analysis. Using the Batch Manager function within CTAn software, the VOIs were thresholded before 3D analysis was performed and a 3D model was generated. All images were viewed and edited using CTVol visualisation software (Bruker). In each VOI the following trabecular morphometric indices were assessed: Bone volume (mm^3), percent bone volume, tissue surface (mm^2), bone surface (mm^2), intersection surface (mm^2), bone surface/volume ratio (1/mm), bone surface density (1/mm), trabecular pattern factor (1/mm), structure model index, trabecular thickness (1/mm), trabecular number (1/mm), trabecular separation (1/mm).

Statistical analysis

Comparisons between two groups were performed using the Student t test or Kruskal-Wallis test (non-parametric) and between multiple groups using ANOVA analysis. The fractional product method [3] was used to discriminate between synergistic and antagonistic effects of drug combinations with a value of > 0.1 indicating antagonism and ≤ -0.1 indicating synergy. The effects of drug interactions were further evaluated by application of the combinatorial index using CompuSyn software (Paramus, NJ) with values < 1 indicating synergy. A multiple linear regression model was used to compare end-of-treatment differences in disease burden between groups of mice with inclusion of covariates to adjust for differences in sex and disease burden prior to commencing treatment (baseline). For survival studies, Kaplan–Meier analysis was performed and a log-rank test used to determine significance. Test statistics resulting in a P-value less than 0.05 were deemed significant. Statistical analyses were performed using Stata version 14 (StataCorp, College Station, TX).

SUPPLEMENTAL REFERENCES

1. Reme T, Hose D, Theillet C, Klein B. Modeling risk stratification in human cancer. doi: 10.1093/bioinformatics/btt124.

Rème T, Hose D, Theillet C, Klein B. Modeling risk stratification in human cancer. Bioinformatics. 2013; 29:1149–57.

2. Pitman MR, Powell JA, Coolen C, Moretti PA, Zebol JR, Pham DH, Finnie JW, Don AS, Ebert LM, Bonder CS, Gliddon BL, Pitson SM. A selective ATP-competitive sphingosine kinase inhibitor demonstrates anti-cancer properties. Oncotarget. 2015; 6:7065–83. doi: 10.18632/oncotarget.3178

3. Webb J. (1963). Effect of more than one inhibitor. In: Hochster R and Quastel J, eds. Enzymes and metabolic inhibitors. (New York, USA: Academic Press), pp. 487-512. 17

4. Fellmann C, Hoffmann T, Sridhar V, Hopfgartner B, Muhar M, Roth M, Lai DY, Barbosa IA, Kwon JS, Guan Y, Sinha N, Zuber J. An optimized microRNA backbone for effective single-copy RNAi. Cell Reports. 2013; 5:1704–13.

5. Liu K, Guo TL, Hait NC, Allegood J, Parikh HI, Xu W, Kellogg GE, Grant S, Spiegel S, Zhang S. Biological characterization of 3-(2-amino-ethyl)-5-[3-(4-butoxyl-phenyl)-propylidene]-thiazolidine-2,4-dione (K145) as a selective sphingosine kinase-2 inhibitor and anticancer agent. PLoS One. 2013; 8:e56471.

6. French KJ, Zhuang Y, Maines LW, Gao P, Wang W, Beljanski V, Upson JJ, Green CL, Keller SN, Smith CD. Pharmacology and antitumor activity of ABC294640, a selective inhibitor of sphingosine kinase-2. J Pharmacol Exp Ther. 2010; 333:129–39.

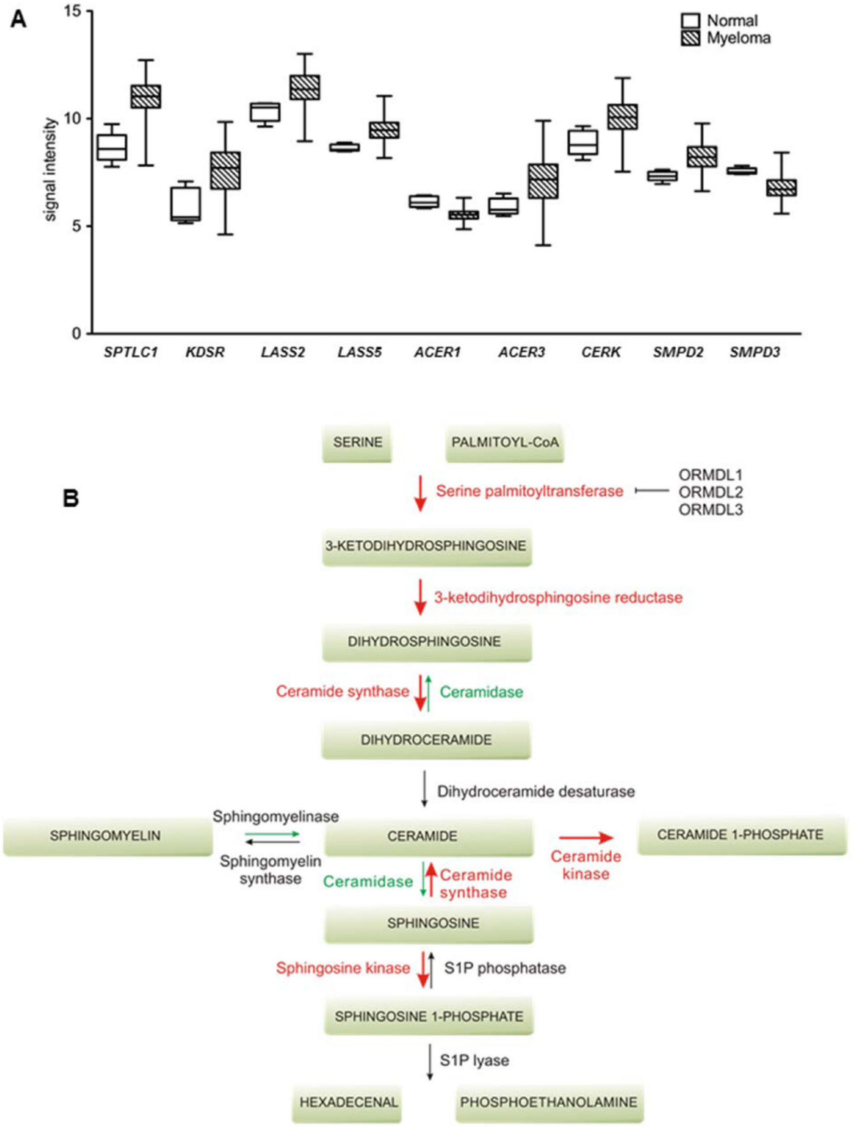

Supplementary Figure 1: Dysregulated sphingolipid enzymes in myeloma patients. (A) Sphingolipid enzymes that show significantly altered (P<0.01) gene expression between normal and MM patients (E-MTAB-363) [1]. (B) Schematic showing the locations of the dysregulated sphingolipid enzymes in (A). Reactions and enzymes shown in red represent where at least one of these genes are up-regulated in myeloma. Reactions and enzymes shown in green represent where some of these genes are up-regulated and others down-regulated in myeloma.

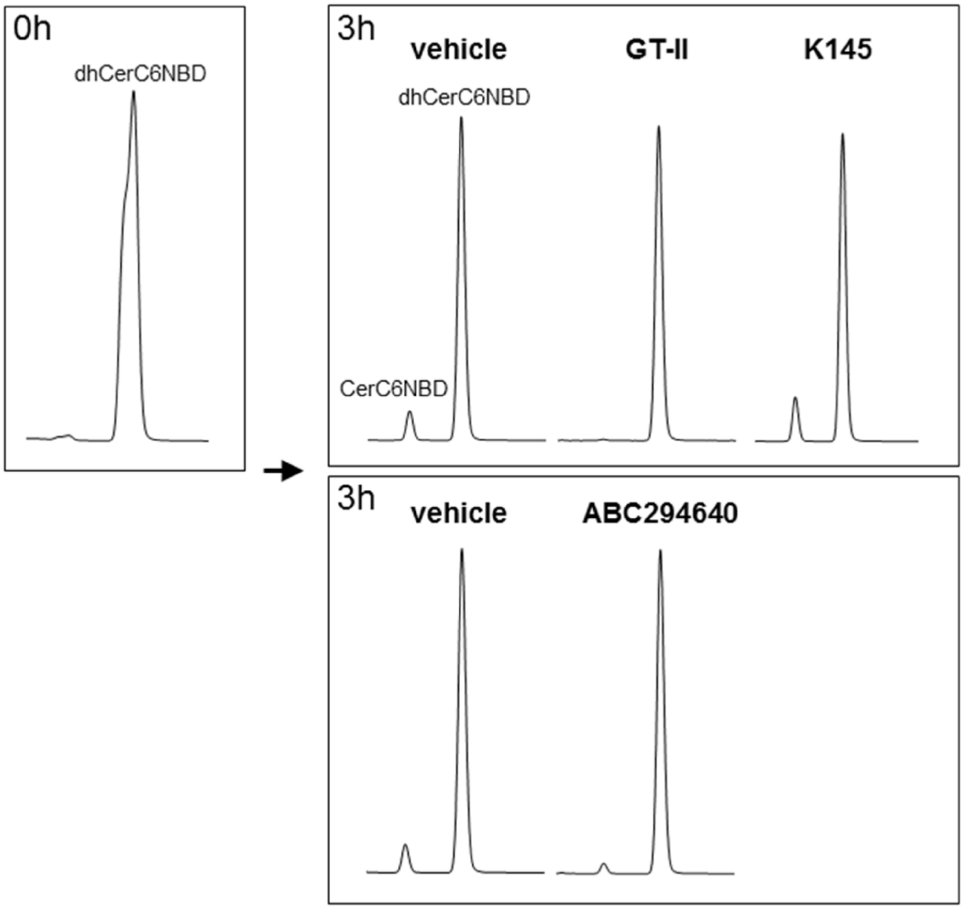

Supplementary Figure 2: K145 does not inhibit dihydroceramide desaturase. The effect of K145 (10 μM), ABC294640 (50 μM) and the dihydroceramide desaturase inhibitor GT-II (1 μM) on dihydroceramide desaturase activity was assessed in intact cells (LP1, upper panel; Jurkat, lower panel) by following conversion of fluorescently labelled C6-dihydroceramide (dhCerC6NBD; Cayman Chemical) to C6-ceramide (CerC6NBD), as previously described [2]. While both GT-II and ABC294640 effectively blocked CerC6NBD generation, no effect was observed with K145. All data shown are representative of three independent experiments.

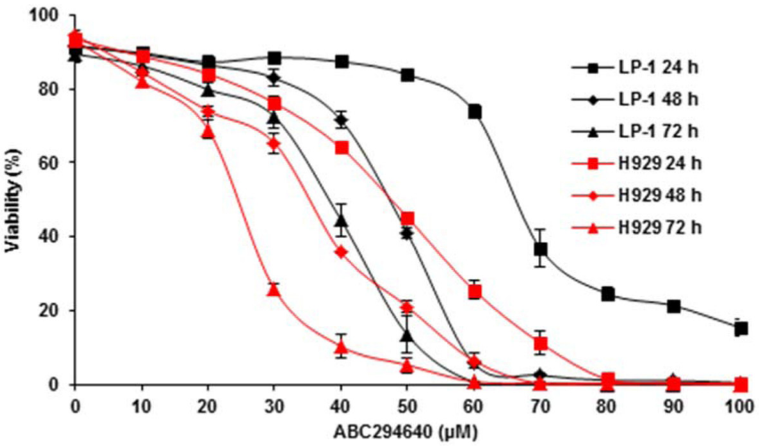

Supplementary Figure 3: ABC294640 synergises with bortezomib to kill myeloma cells. LP-1 and H929 cells were cultured with increasing concentrations of ABC294640 for 24, 48 or 72 h and cell viability measured by flow cytometry. Data are mean±SD of duplicate measurements and are representative of three independent experiments.

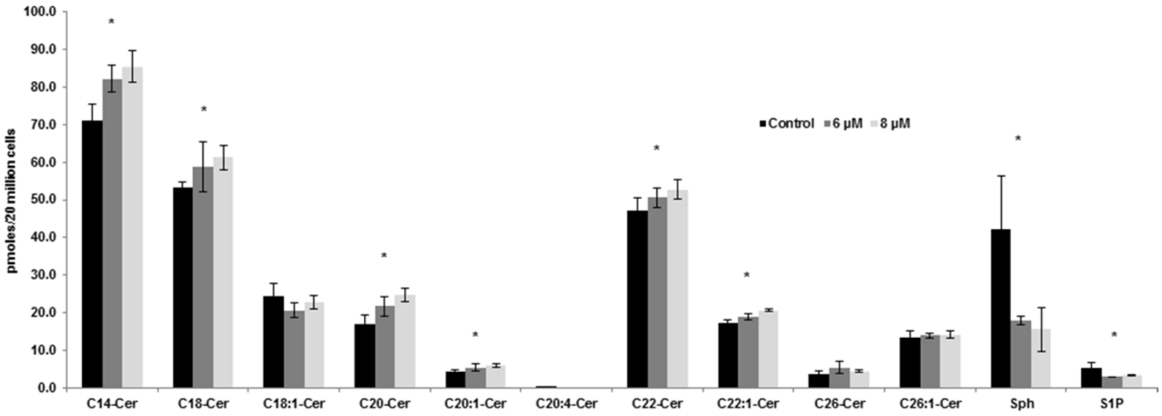

Supplementary Figure 4: K145 induces ceramide accumulation. LP-1 cells were treated with the indicated concentrations of K145 for 6 h and lysates subjected to mass spectrometry for analysis of ceramide (Cer) species, sphingosine (Sph) and sphingosine 1-phosphate (S1P). Mean±SD of quadruplicate measurements shown. * P<0.05.

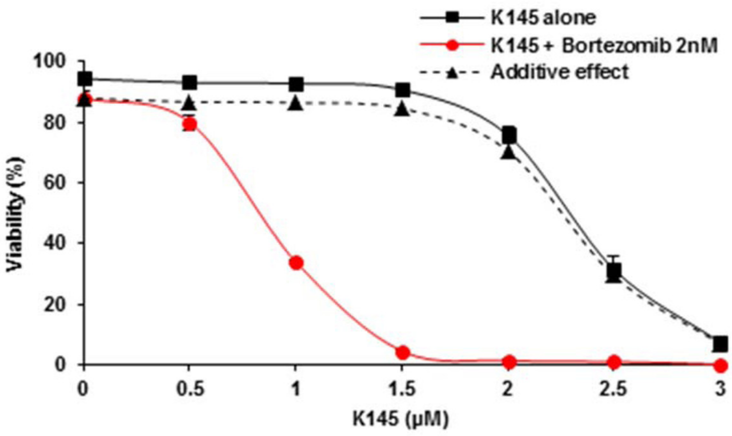

Supplementary Figure 5: K145 synergises with bortezomib in H929 cells. Cells were cultured with the indicated concentrations of K145 with and without 2 nM bortezomib for 24 h and cell viability measured by flow cytometry using Annexin-V/PI staining. Dual negative cells were considered viable. Predicted additive effects, calculated by the fractional product method [3], are shown by the dashed lines and observed combinational effects by the red line. Mean±SD of duplicate measurements shown. Data are representative of three independent experiments.

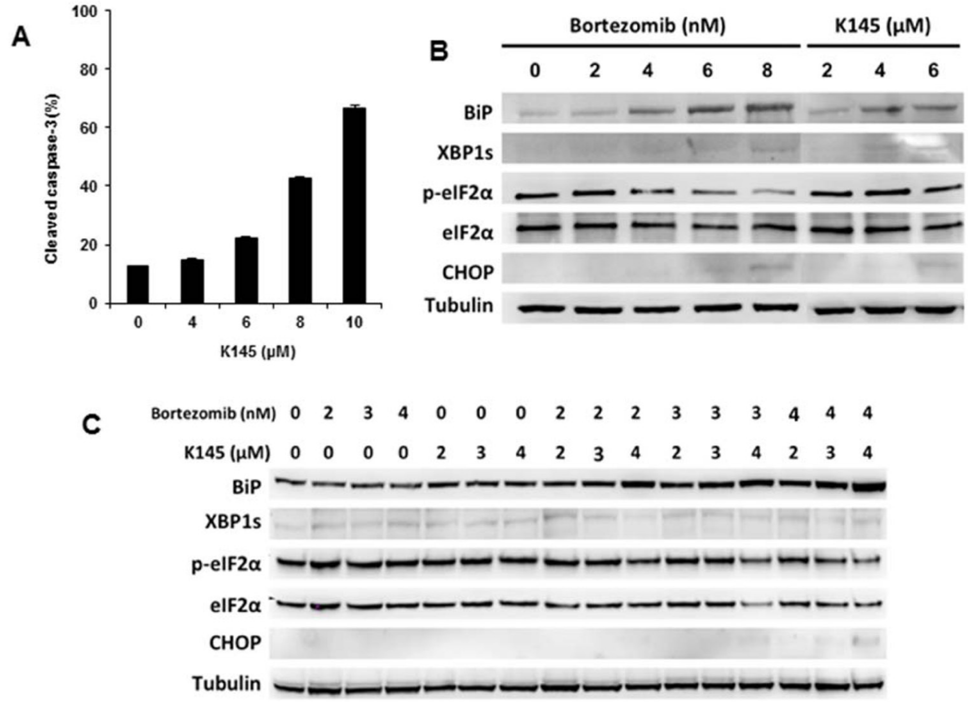

Supplementary Figure 6: Bortezomib and K145 induce synergistic ER stress and the UPR in murine 5TGM1 myeloma cells (A) Cells were treated with the indicated concentrations of K145 for 24 h and after fixation/permeabilisation, levels of cleaved caspase-3 were assessed by flow cytometry. Mean±SD of duplicate measurements shown. 5TGM1 cells were cultured with the indicated concentrations of either bortezomib or K145 (B) and the combination of the two drugs (C) for 16 h and levels of ER stress and UPR proteins determined by Western blot.

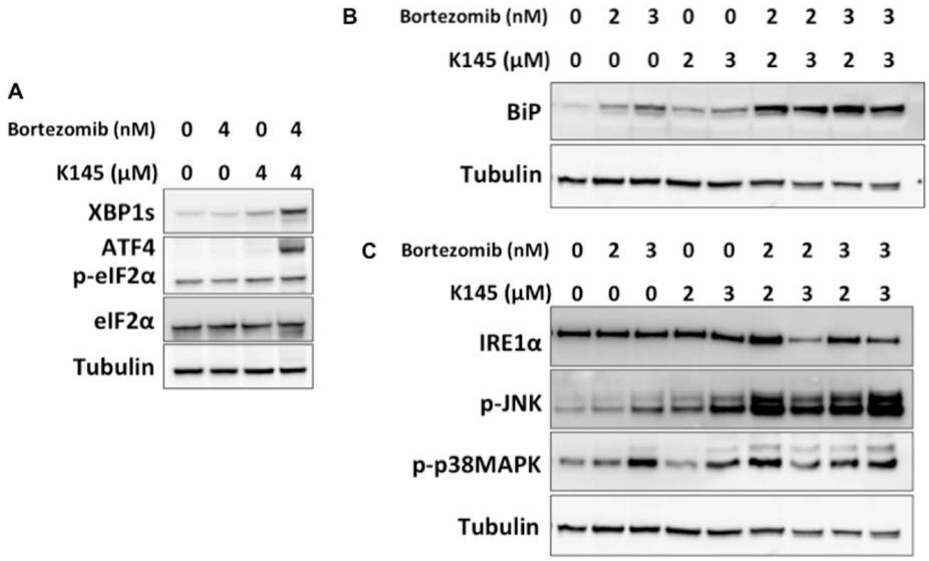

Supplementary Figure 7: Bortezomib and K145 induce synergistic ER stress and UPR activation in OPM2 cells. Cells were cultured with the indicated concentrations of bortezomib and/or K145 for (A) 4 h and (B) 16 h and protein lysates obtained to determine levels of ER stress and UPR components. (C) Protein lysates of OPM2 cells cultured with bortezomib, K145 and the combination for 16 h were examined by Western blot for activation of stress kinases.

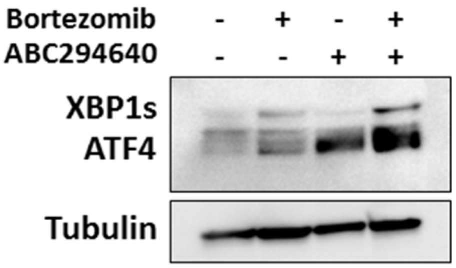

Supplementary Figure 8: Bortezomib and ABC294640 induce synergistic ER stress and UPR activation in LP-1 cells. Cells were cultured with 5 nM bortezomib and/or 20 µM ABC294640 for 4 h and protein lysates obtained to assess ER stress and UPR activation by Western blot.

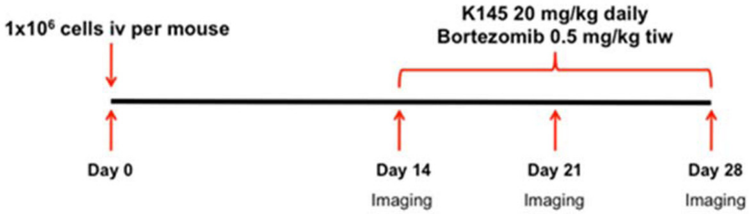

Supplementary Figure 9: Schema of a typical in vivo experiment. Mice are injected with 1 x 106 5TGM1 cells on day 0 and after 14 days are treated with either vehicle (controls), bortezomib 0.5 mg/kg IP thrice weekly, K145 20 mg/kg IP daily or the combination of both drugs for a further 14 days.

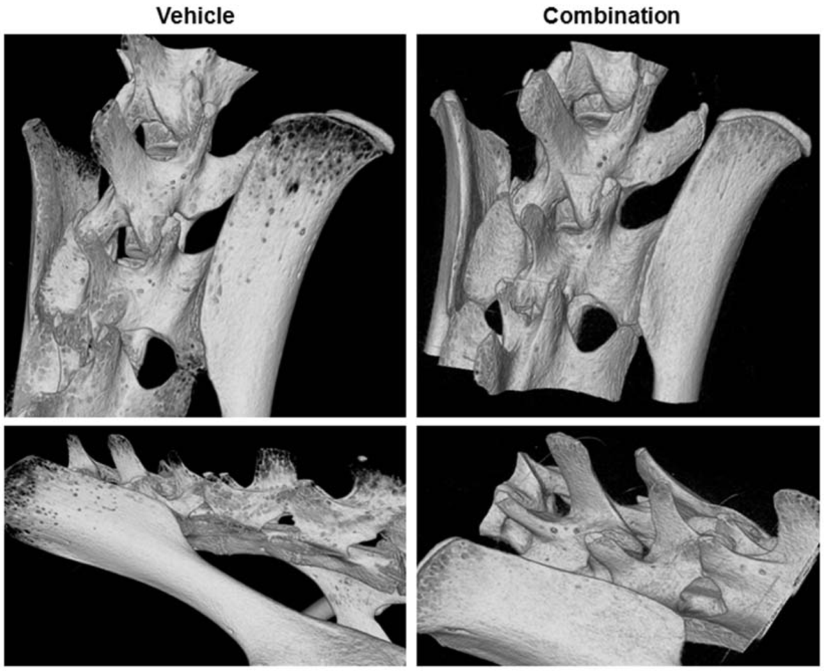

Supplementary Figure 10: Bortezomib and K145 combination therapy results in reduced bone lytic lesions. Representative micro CT images of pelvises from vehicle treated mice (left) and from mice that received the combination of bortezomib and K145 for two weeks (right).

Supplementary Table 1: K145 and bortezomib drug interaction analyses for data shown in Figure 3A, 3C, 3D and Supplementary Figure 5.

LP-1						H929			5TGM1		
K145 (μM)	FP	CI	ABC294640 (μM)	FP	CI	K145 (μM)	FP	CI	K145 (μM)	FP	CI
1	-0.02	1.09	10	-0.06	1.18	0.5	-0.07	0.81	1	-0.32	0.29
2	-0.10	0.7	20	-0.28	1.04	1	-0.56	0.39	2	-0.70	0.28
3	-0.19	0.49	30	-0.41	1.05	1.5	-0.85	0.22	3	-0.82	0.25
4	-0.31	0.32	40	-0.45	0.98	2	-0.73	0.18	4	-0.76	0.24
5	-0.52	0.13	50	-0.25	1.00	2.5	-0.30	0.2	5	-0.59	0.23
6	-0.64	0.05	60	-0.04	0.53	3	-0.07	0.1	6	-0.38	0.2
7	-0.66	0.03	70	-0.02	0.43				7	-0.21	0.21
8	-0.52	0.01	80	-0.01	0.55				8	-0.13	0.19
			90	-0.01	0.62				9	-0.07	0.19
			100	0.00	0.68				10	-0.03	0.21

The indicated myeloma cell lines were cultured with increasing concentrations of K145 or ABC294640 (LP-1 cells) for 24 hours with or without 6 nM bortezomib (LP-1 cells), 2 nM bortezomib (H929 cells) and 4 nM bortezomib (5TGM1 cells). FP, fractional product (FP < -0.1 indicates a synergistic interaction). CI, combinatorial index (CI < 1 indicates a synergistic interaction).

Supplementary Table 2: Bortezomib and K145 drug interaction analysis for data shown in Figure 3B.

LP-1		
Bortezomib (nM)	FP	CI
1	-0.53	0.37352
2	-0.80	0.40228
3	-0.74	0.39465
4	-0.24	0.4829
5	-0.06	0.52674
6	-0.02	0.57543
7	-0.01	0.66361
8	-0.01	0.74055
9	0.00	0.80097
10	0.00	0.94724

LP-1 cells were cultured with increasing concentrations of bortezomib for 24 hours with or without 4 μM K145. FP, fractional product (FP < -0.1 indicates a synergistic interaction). CI, combinatorial index (CI < 1 indicates a synergistic interaction).
